# Emerging therapies for the treatment of hepatitis C

**DOI:** 10.1002/emmm.201303131

**Published:** 2013-09-16

**Authors:** Christian M Lange, Ira M Jacobson, Charles M Rice, Stefan Zeuzem

**Affiliations:** 1 Medizinische Klinik 1, Klinikum der J. W. Goethe-Universität Frankfurt a.M.Frankfurt a.M, Germany; 2 Weill Cornell Medical CollegeNew York, NY, USA; 3 Laboratory of Virology and Infectious Diseases, Center for the Study of Hepatitis C, The Rockefeller UniversityNew York, NY, USA

**Keywords:** directly acting antiviral agents, hepatitis C virus, interferon-free, NS3-4A protease inhibitor, NS5B polymerase inhibitor

## Abstract

Opportunities to treat infection with hepatitis C virus (HCV) are evolving rapidly. From the introduction of interferon-α monotherapy in 1992 to the approval of telaprevir- and boceprevir-based triple therapies with pegylated interferon-α and ribavirin in 2011, the chances of curing patients infected with HCV genotype 1 have improved from <10% to approximately 70%. Significant further improvements are on the horizon, which may well cure virtually all hepatitis C patients with an all-oral, interferon-free regimen in the very near future. These exciting developments are reviewed in the present article.

## The global burden of chronic hepatitis C

Chronic hepatitis C is a highly prevalent infection in global populations affecting approximately 170 million individuals world-wide (Nature Outlook, 2011). Infection with hepatitis C virus (HCV) is frequent in parts of Africa and Asia, with Europe and America considered as regions with a lower prevalence of chronic hepatitis C (0.5–2%) (Nature Outlook, 2011). However, only a minority of those carrying HCV are aware of their infection and can take advantage of treatment options to minimize disease and avoid the risk of further virus transmission (Vermehren *et al*, 2012). This is underscored by the results of a recent study, which indicate that improved screening for HCV infection, in particular for the baby boomer generation, would be cost-effective for preventing HCV-related morbidity (Rein *et al*, 2012).

HCV displays enormous genomic sequence variability due to highly efficient replication resulting in an estimated 10^12^ virions per day coupled with the low fidelity of the HCV RNA polymerase. There exist at least six HCV genotypes as well as multiple subtypes, which differ in ≥30 and ≥10% of their RNA sequence, respectively (Moradpour *et al*, 2007). HCV genotype has a relevant impact on the natural course of hepatitis C. In addition, the chance of treatment-induced virus clearance can be profoundly influenced by genotype and for genotype 1 infection, even by subtype (*e.g*. 1b *vs*. 1a).

A profound inter-individual variability is seen for both the progression of chronic hepatitis C-associated disease as well as treatment outcome. Approximately, 70–80% of patients with acute hepatitis C will develop a chronic infection, where HCV employs multiple strategies to undermine the host's innate and adaptive antiviral immune responses (Thimme *et al*, 2012). A striking example is the cleavage (and inactivation) of MAVS (also called Cardif, VISA and IPS-1) by the HCV NS3-4A serine protease (Schoggins & Rice, 2013). MAVS is an adapter molecule in the retinoic inducible gene I (RIG-I) signaling pathway, which leads to RNA virus induction of type I interferons (Schoggins & Rice, 2013). In addition to viral factors, host genetics play a substantial role in the progression from acute to chronic hepatitis C. As described below more in detail, favourable genetic variations near the *interleukin-28B* (*IL28B*) gene increase the chance of spontaneous clearance from HCV infection by about twofold (Lange & Zeuzem, 2011).

As for the outcome of acute hepatitis C, profound variability exists in the progression of chronic hepatitis C to disease milestones such as the transition from liver fibrosis to cirrhosis or the development of hepatocellular carcinoma (HCC) (Nature Outlook, 2011). Usually, hepatitis C progresses slowly. Liver cirrhosis often occurs only after 20–50 years of infection (Nature Outlook, 2011). Since the incidence of acute hepatitis C has significantly declined during the last decades, the HCV infected population is aging, at least in the Western world. Hence, the number of HCV-infected individuals presenting with advanced liver diseases is on the increase with an expected peak of cases of decompensated liver cirrhosis and HCC around 2020 in the United States (Davis *et al*, 2010). This development will certainly lead to a high demand for HCV-related liver transplantation, accompanied by the enormous task of locating and allocating sufficient liver grafts. To prevent these unfavourable endpoints, improved screening and effective treatment strategies are of major relevance, especially since efforts to develop a vaccine against HCV infection are still in their infancy (Nature Outlook, 2011).

## Classical and emerging therapies for chronic hepatitis C

After discovery of hepatitis A virus (HAV) and hepatitis B virus (HBV) as infectious agents causing viral hepatitis (Alter *et al*, 1978; Tabor *et al*,^1978^), it became evident that many cases of chronic viral hepatitis were not caused by these viruses but rather by an as yet unspecified agent. For this form of hepatitis, the term non-A, non-B hepatitis was coined. HCV was finally discovered after extensive research in 1989 (Choo *et al*,^1989^). Subsequently, infection with HCV explained the majority of cases of non-A, non-B hepatitis, especially in cases related to blood transfusion and injection drug use.

In the first two decades after the discovery of the virus, the evolution of treatment of chronic hepatitis C was driven predominantly by clinical research from the bedside. Even before the discovery of HCV, non-A, non-B hepatitis was treated in a small trial with recombinant interferon-α (IFN-α) (Hoofnagle *et al*, 1986). Although IFN-α in early trials in patients with non-A, non-B hepatitis had striking effects on serum aminotransferase levels, retrospective analyses after the discovery of HCV revealed that only very few patients had achieved a sustained virologic response (SVR, defined by undetectable HCV RNA 24 weeks after treatment completion), which is currently considered an indicator of cure from HCV infection. Subsequent trials after the discovery of HCV yielded SVR rates of 5–20% for treatment with standard IFN-α (reviewed in (Hoofnagle, 2009). As a consequence, IFN-α was approved in the United States in 1992 for the treatment of hepatitis C.

Ribavirin is a nucleoside analogue with activity against a number of viruses. The mechanism(s) of action of ribavirin are complex and not completely understood. Possibilities include direct inhibition of HCV, an increased rate of mutagenesis resulting in a so called “error catastrophe”, inhibition of inosine-monophosphate dehydrogenase (IMPDH), or modulation of innate and adaptive antiviral immune responses including an augmented induction of interferon-stimulated genes in response to IFN-α (Feld & Hoofnagle, 2005; Thomas *et al*, 2011). Although ribavirin alone only had little effect on HCV RNA levels and moderate effects on aminotransferase serum levels, the addition of ribavirin to IFN-α improved SVR rates by more than twofold compared to IFN-α alone (Di Bisceglie *et al*, 1992; McHutchison *et al*, 1998; Poynard *et al*, 1998). Hence, the implementation of ribavirin, which was approved for the treatment of chronic hepatitis C in 1998, represented the next major breakthrough in combating this disease.

The third significant improvement in chronic hepatitis C therapy was the approval of pegylated IFN-α (PEG-IFN-α) in 2001. Pegylation of IFN-α results in profound pharmacokinetic changes characterized by higher and longer-lasting serum concentrations, with the goal of maintaining more constant antiviral pressure on HCV and less frequent dosing. Treatment of chronic hepatitis C with PEG-IFN-α and ribavirin for 48 weeks resulted in SVR rates of 54–56%, compared to 40–50% after treatment with standard IFN-α and ribavirin (Fried *et al*, 2002; Manns *et al*, 2001; Zeuzem *et al*, 2000).

Importantly, SVR rates differ significantly between patient populations. One of the most important predictors of SVR is HCV genotype. While combination therapy with PEG-IFN-α plus ribavirin for 48 weeks results in SVR in only approximately 40–50% of all HCV genotype 1 infected patients, even 24 weeks of PEG-IFN-α/ribavirin therapy usually lead to eradication of HCV genotype 2 or 3 isolates in 70–90% of patients (Hadziyannis *et al*, 2004). In addition to HCV genotype, host determinants such as race, presence of the metabolic syndrome, or presence of advanced liver fibrosis are important predictors of treatment failure (Berg *et al*, 2003; Lange *et al*, 2012). In this regard, the recent identification of genetic variations near *IL28B* by four independent genome-wide association studies (GWAS) as strong predictors of treatment-induced clearance from HCV infection represented an unexpected breakthrough (Lange & Zeuzem, 2011). In detail, a good-response *IL28B* genotype identifies HCV genotype 1 patients with an almost doubled chance to be cured by PEG-IFN-α/ribavirin therapy in contrast to those patients with an unfavourable *IL28B* genotype. Importantly, a subsequent study has led to the discovery of a novel gene encoding IFN-λ4 (IFNL4), which is only present in individuals carrying the poor response *IL28B* genotype, and appears to alter the expression of interferon-stimulated genes (Prokunina-Olsson *et al*, 2013).

As indicated above, PEG-IFN-α/ribavirin therapy has been developed empirically. In contrast, the development of directly acting antiviral agents (DAAs) required a better understanding of viral structures and the viral life cycle. An important breakthrough was the development of subgenomic HCV replicons and—subsequently—full-length clones of HCV which are infectious in cell culture [cell-culture derived HCV, HCVcc] (Lindenbach *et al*, 2005; Lohmann *et al*, 1999; Wakita *et al*, 2005), which led to an explosive understanding of the HCV life cycle. In addition, the structural features of several HCV proteins could be resolved by intensive research (Bartenschlager *et al*, 2013). As a consequence, numerous DAAs, which specifically target essential viral functions, have been developed in the last decade by intensive bench to bedside research (Lange & Zeuzem, 2012). Furthermore, an increasing number of host factors such as cyclophilin A or miR-122 (see below) which are required for HCV replication have been identified as promising candidates for host-targeting antiviral agents (HTAs). Results of numerous clinical studies clearly indicate that the implementation of DAAs (and perhaps HTAs) will revolutionize therapy of chronic hepatitis C (Lange & Zeuzem,^2012^). The HCV NS3-4A protease inhibitors telaprevir and boceprevir were first to complete clinical development and were approved in the US and Europe in 2011. In combination with PEG-IFN-α and ribavirin these protease inhibitors are currently the standard of care for treatment of HCV genotype 1 infection (details below). This advance in therapy occurred nearly contemporaneously with proof of concept studies showing that HCV can be eradicated without interferon. These developments may well lead to the possibility of curing the vast majority of hepatitis C patients with all-oral, IFN-free regimens in the very near future, *i.e*. about 30 years after the discovery of HCV.

## The HCV life cycle and targets for directly acting antiviral therapy

### HCV life cycle

HCV, a member of the *Flaviviridae* family, has a positive-sense single-stranded RNA genome of approximately 9600 nucleotides encoding a polyprotein of more than 3000 amino acids (Moradpour *et al*, 2007). The HCV polyprotein contains the structural proteins core, E1 and E2, as well as the non-structural proteins p7, NS2, NS3, NS4A, NS4B, NS5A and NS5B. In addition, the HCV genome contains conserved HCV 5′ and 3′ untranslated regions (UTRs). The 5′UTR is of particular importance as it is structured in four domains building the internal ribosome entry site (IRES), a structure that is mandatory for proper initiation of HCV RNA translation (Niepmann, 2013).

Together with host-derived lipids, the structural HCV proteins core, E1 and E2 form the viral particle, which binds to cellular receptors for viral attachment and host cell entry (Moradpour *et al*, 2007). Key cellular receptors for HCV entry are CD81, claudin-1, occludin, scavenger receptor BI, and others (Zeisel *et al*, 2013). Binding of HCV to its cellular receptors is followed by receptor-mediated endocytosis and viral uncoating, which leads to the release of the HCV genome into the cytoplasm to serve as a messenger RNA for the HCV polyprotein precursor. Complex formation of the IRES with the 40S ribosomal subunit, eucaryotic initiation factors and viral proteins then initiates HCV mRNA translation (Niepmann, 2013). From the initially translated HCV polyprotein, the three structural and seven non-structural HCV proteins are processed by both host and viral proteases. NS2 is a cysteine protease with unique structural features that mediates a single cleavage at the NS2/NS3 junction, leading to autoinhibition and generation of the NS3 N terminus (Lorenz *et al*, 2006). NS3 contains a serine protease domain as well as a helicase/NTPase domain. The serine protease domain is composed of two β-barrels and four α-helices (Kim *et al*, 1996). The serine protease catalytic triad—histidine 57, asparagine 81 and serine 139—is located in a small groove between the two β-barrels. NS3 catalytic activities depend on formation of a tight, non-covalent complex with the HCV NS4A protein, a cofactor mandatory for proper protein folding and stabilization of the protease in proximity of cellular lipid membranes (Lindenbach, 2013; Lohmann, 2013). The NS3-4A protease cleaves the junctions between NS3/NS4A, NS4A/NS4B, NS4B/NS5A and NS5A/NS5B (Morikawa *et al*, 2011). In addition to HCV polyprotein procession, NS3 is required for HCV RNA replication, perhaps involved in unwinding of nascent viral RNA by its helicase activity. In addition, NS3 is required for efficient viral assembly and particle production (Lindenbach, 2013).

Polyprotein processing and formation of the RNA replication complex is required for HCV RNA replication. The HCV replication complex is composed of viral proteins and RNA and cellular proteins, which associate with rearranged intracellular membranes (Lohmann, 2013). These rearranges cellular membranes are derived mainly from the endoplasmic reticulum and form accumulations of single and double membrane vesicles in a structure called the membranous web. Formation of the membranous web is important for a well-coordinated organization of HCV replication and may furthermore help HCV to escape antiviral immunity. The HCV NS5B protein is an RNA-dependent RNA polymerase that executes HCV replication by producing complementary negative-stranded RNAs to the positive-stranded HCV RNA genome (Lohmann, 2013). Subsequently, these negative-stranded RNAs serve as templates to catalyse the synthesis of novel positive-stranded HCV RNA genomes. A poor fidelity and a high error rate are cardinal features of NS5B. These—combined with a high flexibility of HCV to tolerate many mutations without relevant loss of viral fitness—promote the high genetic variability of HCV, which can lead to the generation and rapid selection of drug-resistant variants during therapy with some DAAs.

NS4B and NS5A also play essential roles in HCV RNA replication. NS4B is a hydrophobic protein that oligomerizes and cooperates with other non-structural HCV proteins (especially NS5A) to induce formation of the membranous web, which serves as a scaffold for the HCV replication complex (Lohmann, 2013; Romero-Brey *et al*, 2012). The NS5A protein plays many roles in HCV replication, assembly and release. An interaction of NS5A with intracellular lipid membranes is of particular importance for the formation of the HCV RNA replicase (Bartenschlager *et al*, 2004). Furthermore, NS5A has RNA binding properties, which may protect or direct newly synthesized HCV RNA for different fates (translation, replication or packaging) (Lohmann, 2013). In addition, NS5A promotes virus assembly in the proximity of lipid droplets, a process for which an interaction between NS5A and the core protein appears to play an essential role (Lindenbach, 2013). Finally, NS5A appears to enhance the activity of NS5B (Lohmann, 2013). In addition to these viral proteins, cellular proteins such as cyclophilin A are essential co-factors of HCV RNA replication. Interestingly, HCV RNA replication also depends crucially on a liver-specific micro-RNA (miR-122), which may partially explain the hepatotropism of HCV (Landford *et al*, 2010).

Viral assembly and release takes place in close association to lipid droplets, probably by usage of the host's lipoprotein particle production pathway. As indicated above, NS5A and core are key players in the translocation of viral structures from the replication complex to the assembly machinery to lipid droplets, but apparently all viral proteins are involved in the complex process of viral morphogenesis and secretion (Lindenbach, 2013).

**Figure 1:**
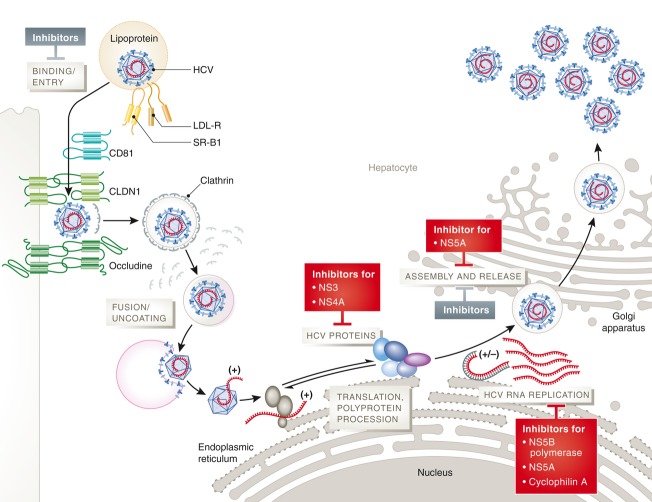
Major steps of the HCV life cycle are shown schematically. HCV polyprotein procession is targeted by NS3-4A inhibitors. HCV RNA replication is targeted by NS5B polymerase inhibitors, NS5A inhibitors, and cyclophilin A inhibitors. However, HCV proteins interact throughout the HCV life cycle, and DAAs therefore impact on multiple steps in the HCV life cycle. This was shown for example for NS5A inhibitors, which affect HCV replication, assembly and release. Specific inhibitors of viral entry or assembly and release are in preclinical or early clinical development. CLDN1, claudin-1; LDL-R, low density lipoprotein receptor; SR-B1, scavenger receptor B1.

In principle, each of the HCV proteins, HCV-specific RNA structures such as the IRES, as well as host factors on which HCV depends, are suitable targets for DAAs/HTAs (Fig 1). The most successful DAAs, which have been developed in the last years target the HCV protease NS3-4A, the NS5B polymerase, and the NS5A protein (Table 1).

**Table 1:** Cardinal features of DAAs and HTAs

	HCV genotype coverage	Antiviral activity	Barrier to resistance development
NS3-4A inhibitors	Narrow to broad	High	Low
Nucleoside NS5B inhibitors	Broad	Intermediate to high	High
Non-nucleos(t)ide NS5B inhibitors	Narrow	Low to intermediate	Low
NS5A inhibitors	Broad	High	Low
HTAs (Cyclophilin A or miR-122 inhibitors)	Broad	Intermediate to high	High

### NS3-4A protease inhibitors

The first NS3-4A inhibitor, which was investigated in patients with hepatitis C was ciluprevir (BILN 2061), an orally bioavailable, peptidomimetic, macrocyclic drug binding non-covalently to the active centre of the protease (Lamarre *et al*, 2003). Ciluprevir monotherapy resulted in a profound and rapid reduction of HCV RNA serum levels in HCV genotype 1 patients, which served as a proof-of-concept that DAAs can inhibit HCV in infected individuals. Yet, ciluprevir development was stopped due to cardiotoxicity. In the meantime, numerous other NS3-4A protease inhibitors have been developed which belong to two different molecular classes, the macrocyclic inhibitors and linear tetra-peptide α-ketoamide derivatives. As ciluprevir, first generation NS3-4A inhibitors display potent antiviral activity, but also a low barrier to resistance development. Hence, monotherapy with NS3-4A inhibitors can result in an approximately 4 log_10_ decrease of serum HCV RNA within days, but also in rapid selection of resistant variants and viral breakthrough (Lange & Zeuzem, 2012). Both addition of PEG-IFN-α/ribavirin or of other DAAs can successfully reduce the frequency of resistance development (Bacon *et al*, 2011; Jacobson *et al*, 2011; Poordad *et al*, 2011; Zeuzem *et al*,2011a). The NS3-4A inhibitors telaprevir and boceprevir were approved in 2011. Other NS3-4A inhibitors are in various phases of clinical evaluation (*e.g*. simeprevir (TMC435), faldaprevir (BI201335), danoprevir (R7227/ITMN191), vaniprevir (MK-7009), asunaprevir (BMS-650032), ABT-450, MK-5172, or GS-9256). Their advantages over telaprevir/boceprevir are improved pharmacokinetics with the capacity for less frequent dosing, broader genotypic activity in some cases and, most importantly, a lower frequency of side-effects (Lange & Zeuzem, 2012).

### NS5B polymerase inhibitors

NS5B RNA polymerase inhibitors can be categorized into two different classes, nucleos(t)ide analog inhibitors (NIs) and non-nucleos(t)ide inhibitors (NNIs). After intracellular phosphorylation, NIs like mericitabine (R7128) or sofosbuvir (GS-7977) get incorporated into the growing RNA chain as false substrates, which causes direct chain termination. NIs are usually effective against all HCV genotypes since the active centre of NS5B is a highly conserved region. Furthermore, this feature of the polymerase conditions a high barrier to resistance against NIs given the low fitness of resistant variants. Valopicitabine, the first NI investigated in patients with chronic hepatitis C, and other early NIs showed only low to moderate antiviral activity (Afdhal *et al*, 2007). The clinical development of valopicitabine as well as of a number of subsequent NIs (*e.g*. R1626, PSI-938, BMS-986094) has been stopped due to a variety of toxicity issues. However, an NI such as sofosbuvir, which combines highly potent antiviral activity, a high barrier to resistance, pangenotypic coverage, and an excellent safety record, identifies the NI class as a compelling cornerstone of upcoming IFN-α-based or IFN-free all-oral combination therapies (Gane *et al*, 2013b).

In contrast to NIs, the more heterogeneous class of NNIs inhibits NS5B activity by binding to different allosteric enzyme sites resulting in conformational changes before the elongation complex is established. NS5B displays classic “right hand” polymerase features with fingers, palm and thumb domains, and at least four NNI-binding sites, a benzimidazole-(thumb 1)-, thiophene-(thumb 2)-, benzothiadiazine-(palm 1)- and benzofuran-(palm 2)-binding site (Bartenschlager *et al*, 2013). NNIs targeting each of these sites have been developed (*e.g*., thumb 1 inhibitors BI207127, BMS-791325; thumb 2 inhibitors filibuvir (PF-00868554) and VX-222; palm I inhibitor ANA598 and ABT-333; palm II inhibitors tegobuvir and IDX-375). Since NNIs do not bind to the highly conserved active centre of NS5B, their resistance barrier is low (Lange & Zeuzem,^2012^). Furthermore, most NNIs studied to date are characterized by low to moderate antiviral activity, and—in contrast to NIs—their genotypic coverage is narrow. Nevertheless, some NNIs are in development as components of non-NI containing interferon-free regimens and have demonstrated quite high levels of efficacy in phase 2 trials (see below).

### NS5A inhibitors

Daclatasvir (BMS-790052), the first NS5A inhibitor evaluated in patients, is a small molecular compound binding to domain I of NS5A (Gao *et al*, 2010). Very low doses of daclatasvir can sufficiently suppress all HCV genotypes both *in vitro* and *in vivo* (Fridell *et al*, 2011). Moreover, the safety profile of many NS5A inhibitors appears to be excellent. These features have brought daclatasvir and other NS5A inhibitors (*e.g*. BMS-824393, PPI-461, GS-5885; ABT-267, IDX-719) into prominent positions in the current development of IFN-α-based and IFN-free clinical trials, although their genetic and fitness barriers to resistance development are low.

### Host-targeting antiviral agents (HTAs)

An increasing number of host factors has been discovered which are mandatory for an intact HCV life cycle. Most of these host factors are cellular proteins such as enzymes, receptors or kinases; but also lipids and at least one micro-RNA (miR-122). Cyclophilin A is a prominent example of a cellular enzyme that enhances HCV replication by incompletely understood mechanisms, including the modulation of NS5B activity (Lohmann, 2013). Alisporivir (Debio-025) is an orally bioavailable cyclophilin A inhibitor exerting a profound antiviral activity on HCV both*in vivo* and *in vitro* (Flisiak *et al*, 2009). Importantly, alisporivir is effective against all HCV genotypes and displays a very high barrier to resistance development. Despite these favourable characteristics, the development of alisporivir is currently on hold due to several cases of pancreatitis during combination therapy with alisporivir and PEG-IFN-α-2a. Nevertheless, clinical trials evaluating alisporivir in both interferon-containing and interferon-free regimens have suggested a potential role for HTAs in HCV treatment regimens (Pawlotsky *et al*, 2012).

miR-122, a liver-specific micro RNA, binds two different sites in the HCV 5′NTR and plays an essential role in HCV replication (Landford *et al*,^2010^). Miravirsen, an antisense oligonucleotide against miR-122 that prevents binding of miR-122 to the HCV 5′NRT, is the first micro-RNA targeting drug evaluated in clinical studies. A Phase IIa proof-of-principle study showed that miravirsen monotherapy can achieve a HCV RNA decline in serum of up to 2.7 log_10_ IU/ml (Janssen *et al*, 2013).

## Therapeutic strategies in the era of DAAs

### IFN-α-based triple- and quadruple therapies

As indicated above, the addition of telaprevir or boceprevir to PEG-IFN-α and ribavirin resulted in significantly higher SVR rates in both treatment-naïve and treatment-experienced HCV genotype 1 patients compared to dual therapy with PEG-IFN-α and ribavirin alone [67–75% *vs*. 40–44% in treatment-naïve patients, 31–86% *vs*. 5–29% in treatment-experienced patients, respectively] (Bacon *et al*, 2011; Jacobson *et al*,^2011^; Poordad *et al*, 2011; Zeuzem *et al*, 2011a). As a consequence, telaprevir- and boceprevir-based triple therapy is currently considered the standard of care for HCV genotype 1 patients. The superiority of triple therapy regimens over PEG-IFN-α and ribavirin alone has been demonstrated with other DAAs such as newer NS3-4A protease inhibitors, NS5A inhibitors, NI NS5B polymerase inhibitors or the HTA alisporivir. For example, SVR rates in treatment-naive HCV genotype 1 patients included in a phase III clinical study (STARTVerso1) were 79–80% after treatment with triple therapy based on the second wave protease inhibitor faldaprevir for 24–48 weeks compared to 52% after dual therapy with PEG-IFN-α/ribavirin (Ferenci *et al*, 2013). Other phase III clinical trials (QUEST-1, QUEST-2) have shown that the NS3-4A inhibitor simeprevir for 12–24 weeks in combination with PEG-IFN-α/ribavirin for 24–48 weeks resulted in overall SVR rates of 80–81% compared to 50% after PEG-IFN-α/ribavirin therapy in treatment-naïve HCV genotype 1 patients (Jacobson *et al*, 2013a; Manns *et al*, 2013). Striking phase 2 results were obtained for triple therapy including the NI sofosbuvir, which resulted in 90% SVR in treatment-naïve HCV genotype 1 patients, and even higher in genotype 2 and 3 patients, after only 12 weeks of treatment (Kowdley *et al*, 2013a; Lawitz *et al*, 2013a). The promise of this regimen was affirmed by a 90% SVR rate in a phase 3 trial in treatment-naïve patients with genotypes 1, 4, 5 and 6 (Lawitz *et al*, 2013b). Triple therapy with the NS5A inhibitor daclatasvir led to SVR in 59–100% of treatment-naïve HCV genotype 1 and 4 patients, according to daclatasvir dosage and HCV genotype/subtype (Pol *et al*, 2012).

Overall, these data exemplarily demonstrate a high potential of the triple therapy approach, which certainly represents an enormous progress compared to dual therapy with PEG-IFN-α and ribavirin alone. Nevertheless, triple therapy has significant limitations on efficacy. Most importantly, SVR rates in specific patient populations after triple therapy are still unsatisfactory. For example, only about 14% of patients with liver cirrhosis and previous null-response to PEG-IFN-α/ribavirin achieved SVR after telaprevir-based triple therapy (Zeuzem *et al*, 2011a). These data clearly demonstrate that the individual responsiveness to IFN-α as well as the stage of liver disease still represents important determinants of outcome of triple therapy. In addition, success of triple therapies differs significantly according to infection with specific HCV subtypes. For example, in all telaprevir-based triple therapy studies viral resistance and breakthrough occurred more frequently in patients infected with HCV genotype 1a compared to genotype 1b (Jacobson *et al*, 2011). This difference was shown to result from nucleotide differences at the predominant position for resistance development (R155) in HCV subtype 1a *versus* 1b (McCown *et al*, 2009).

To overcome limited efficacy of triple therapy in difficult-to-cure patient populations, a so-called quadruple therapy approach including two DAAs of different classes in combination with PEG-IFN-α and ribavirin has been successfully employed. In principle, combining DAA agents with distinct antiviral resistance profiles should significantly reduce the risk of resistant development. Indeed, phase I and II clinical trials evaluating quadruple therapy have revealed outstanding SVR rates even in HCV genotype 1a patients with previous null-response to PEG-IFN-α and ribavirin. For example, in a small but highly informative proof-of-concept study the combination of the NS5A inhibitor daclatasvir and the protease inhibitor asunaprevir together with PEG-IFN-α and ribavirin for 24 weeks resulted in 100% SVR in HCV genotype 1 patients with prior null-response (Lok *et al*, 2012). Additional trials have shown that quadruple therapies including combinations with NNIs and NS3-4A inhibitors (*e.g*. tegobuvir plus GS-9256 or VX-222 plus telaprevir), or with the NI mericitabine and the NS3-4A inhibitor danoprevir also result in high virologic response rates (Feld *et al*, 2012; Jacobson *et al*, 2012; Zeuzem *et al*, 2011c). Though these trials were small and performed in highly selected patients, the quadruple therapy approach appears to be highly promising in patients with limited sensitivity to IFN-α, even in difficult-to-cure patients with HCV subtype 1a and prior null-response to PEG-IFN-α/ribavirin. However, due to the changing epidemiology of the HCV infected population, there is an increasing number of patients who have contraindications or do not tolerate PEG-IFN-α or ribavirin. For these individuals, triple and quadruple therapy approaches are no option. Moreover, even for individuals capable of interferon treatment there is a universal desire to avoid interferon because of its safety and tolerability issues. Therefore, results of numerous recent studies, which have proven that IFN-free combination therapies with combinations of different DAA agents (±ribavirin) can successfully result in viral eradication are of major importance (Table 2).

**Table 2:** Selected trials evaluating IFN-free DAA combination therapies

DAAs combined	Additional medication	Phase	Patient population	SVR rate
Nucleos(t)ide NS5B inhibitor				
Sofosbuvir GS-7977	±Ribavirin	3	GT 1 naiv	84%^a^
			GT 1 experienced	11%^a^
			GT 2 naiv	91–98%
			GT 2 experienced	60–100%
			GT 3 naiv	34–61%
			GT 3 experienced	19–63%
Nucleos(t)ide NS5B inhibitor + NS3-4A inhibitor				
Mericitabine + danoprevir/ritonavir	±Ribavirin	3	GT 1 naiv	26% (1a), 71% (1b)
Sofosbuvir + simeprevir	±Ribavirin	2	GT 1 null-responder	>90%
Nucleosi(t)de NS5B inhibitor + NS5A inhibitor				
Sofosbuvir + daclatasvir	±Ribavirin	2	GT 1 naiv	100%
			GT 2-3 naiv	86–88%
Sofosbuvir + ledispavir	±Ribavirin	3	GT 1 naiv and experienced	>90%
Non-nucleos(t)ide NS5B inhibitor + NS3-4A inhibitor				
BI-207127 + faldaprevir	+Ribavirin	3	GT 1 naiv	39–68%
Tegobuvir + GS-9256	±Ribavirin	2	GT 1 naiv	n.a.
ABT-333 + ABT-450/ritonavir	+Ribavirin	3	GT 1 naiv	93%
			GT 1 null-responder	47%
ABT-072 + ABT-450/ritonavir	+Ribavirin	2	GT 1 naiv	91%
VX-222 + telaprevir	±Ribavirin	2	GT 1 naiv	n.a.
NS3-4A inhibitor + NS5A inhibitor				
Asunaprevir + daclatasvir	±Ribavirin	3	GT 1 null-response	36–78%
Simeprevir + daclatasvir	±Ribavirin	2	GT 1 naiv and experienced	n.a.
Multiple DAA agent combinations				
NS5A-inhibitor (ledispavir) + NS3-4A inhibitor (GS-9451) + NNI (tegobuvir)	+Ribavirin	2	GT 1 naiv	77% (1a), 89% (1b)
NS5A-inhibitor (ABT-267) + NS3-4A inhibitor (ABT-450/r) + NNI (ABT-333)	+Ribavirin	2	GT 1 naiv and experienced	>90%
NS5A-inhibitor (daclatasvir) + NS3-4A inhibitor (asunaprevir) + NNI (BMS-791325)	–	2	GT 1 naiv	90%
Host targeting agents				
Alisporivir	±Ribavirin	3 (on hold)	Selected GT 2, 3	70–90%
Miravirsen	(+PEG-IFN-α and ribavirin)	2	GT 1	n.a.

a These SVR rates are derived from much smaller studies than those evaluating sofosbuvir in HCV genotype 2 and 3 infection.

### All-oral therapy regimens with or without ribavirin

#### Nucleoside analog NS5B inhibitor-based IFN-free regimens, with or without ribavirin

The first IFN-free clinical trial (INFORM-1 study) assessed a dual therapy with the NI mericitabine and the NS3-4A inhibitor danoprevir. HCV RNA declined up to 5.2 log_10_ IU/ml during treatment, which represents a stronger antiviral efficacy of dual therapy compared to monotherapy with each of these agents (Gane *et al*, 2010). Yet, this study did not address the key question whether a cure from HCV infection can be achieved by IFN-free regimens, as PEG-IFN-α and ribavirin rescue therapy was administered to all patients after 14 days of all-oral therapy. However, a follow-up study (INFORM-SVR) showed that SVR rates of 26 and 71% were achieved in treatment-naïve HCV genotype 1a and 1b patients, respectively, who were treated with mericitabine, danoprevir (ritonavir boosted) and ribavirin for 12–24 weeks (Gane *et al*, 2012). SVR rates were consistently lower in all ribavirin-free treatment groups. Importantly, in patients with viral breakthrough resistance associated variants (RAVs) were predominantly identified within NS3-4A while a resistance mutation in NS5B was discovered only in a single patient (S282T).

The NI sofosbuvir is characterized by higher antiviral efficacy than mericitabine. In relatively small phase II trials in treatment-naïve HCV genotype 1, 2 and 3 patients, sofosbuvir plus ribavirin administered for 12 weeks resulted in impressive SVR rates of 84–100%, but only 11% (1/9) of treatment-experienced HCV genotype 1 patients achieved SVR after 12 weeks of therapy with sofosbuvir plus ribavirin (Gane *et al*, 2013b). The large phase III Fission trial compared 12 weeks of sofosbuvir plus ribavirin with 24 weeks of PEG-IFN-α and ribavirin in treatment-naïve HCV genotype 2 and 3 patients. Both treatment arms resulted in overall SVR rates of 67%. However, in patients infected with HCV genotype 3, SVR rates were significantly lower (34 and 61% in patients with or without liver cirrhosis, respectively) than in HCV genotype 2 patients (91 and 98% in patients with or without liver cirrhosis, respectively) (Lawitz *et al*, 2013b). Comparable SVR rates were observed in another phase III trial (Positron) in IFN-intolerant, -ineligible or -unwilling HCV genotype 2 and 3 patients who were treated for 12 weeks with sofosbuvir and ribavirin (Jacobson *et al*, 2013b). Finally, the phase III Fusion trial assessed sofosbuvir plus ribavirin for 12 or 16 weeks in treatment-experienced HCV genotype 2 and 3 patients. In HCV genotype 2 patients with or without liver cirrhosis, SVR rates after 12 weeks were 60 and 96%, respectively, and 78 and 100% after 16 weeks of therapy (Jacobson *et al*, 2013b). However, in HCV genotype 3 patients with or without liver cirrhosis, SVR rates after 12 weeks were only 19 and 37%, respectively, but 61 and 63% after 16 weeks of therapy (Jacobson *et al*, 2013b). Overall, therapy with sofosbuvir plus ribavirin for 12 weeks seems be a potent and promising regimen for HCV genotype 2 patients, but for other HCV genotypes, especially in patients with liver cirrhosis, alternative strategies such as longer treatment durations, or the addition of another DAA or of PEG-IFN-α appear to be necessary.

#### NS5A inhibitors combined with sofosbuvir

Combination therapy with sofosbuvir plus the NS5A inhibitor daclatasvir with or without ribavirin for 12 or 24 weeks yielded impressive results. In approximately 90 treatment-naïve patients, SVR rates were 100 and 86–88% in HCV genotype 1 and HCV genotype 2 and 3 patients, respectively (Sulkowski *et al*, 2012a). In this study, the addition of ribavirin did not improve virologic response rates but led to an increased rate of side effects. Perhaps even more dramatically, the same regimen given for 24 weeks to 41 genotype 1 patients who had failed protease inhibitor-based triple therapy yielded an SVR rate of 100%, with or without ribavirin (Sulkowski *et al*, 2013). In addition, sofosbuvir was evaluated in combination with another NS5A inhibitor (ledispavir, GS-5885) plus ribavirin as well as in combination with the NNI GS-9669 plus ribavirin. In both treatment-naïve and treatment-experienced HCV genotype 1 patients, treatment with these combinations resulted in SVR rates >90% (Gane *et al*, 2013a).

#### NS3-4A inhibitor-based IFN-free regimens, with or without ribavirin

NS3-4A inhibitors have been evaluated in IFN-free trials in combination with NIs but also in combination with NNIs or NS5A inhibitors. The SOUND-C1 and SOUND-C2 trials assessed combination therapy of the NS3-4A inhibitor faldaprevir (BI-201335), the NNI BI-207127 and ribavirin in treatment-naïve HCV genotype 1 patients. In SOUND-C1, virologic response rates for INF-free therapy were up to 82, 100 and 100% at treatment days 15, 22 and 29, respectively (Zeuzem *et al*, 2011b, 2013). After 16–40 weeks of IFN-free therapy, SVR rates in the large SOUND-C2 trial ranged from 56 to 68% in treatment arms including ribavirin, compared to 39% in a single ribavirin-free treatment arm (Zeuzem *et al*, 2012). Within all treatment arms, SVR rates were consistently higher in HCV genotype 1b vs. 1a patients, or in patients with a good-response*IL28B* genotype. Importantly, SOUND-C2 was one of very few studies, which included a significant number of patients with liver cirrhosis. In these patients, SVR rates were still promising, though lower than in patients without advanced liver disease. Relatively low on-treatment virologic response rates were observed in two other studies evaluating NS3-4A inhibitors in combination with NNIs ±ribavirin [GS-9256 + tegobuvir (Zeuzem *et al*, 2011c); telaprevir + VX-222 (Jacobson *et al*, 2012)]. These data for all-oral combinations based on NS3-4A inhibitors plus NNIs were supplemented by strongly encouraging results of the recent Co-Pilot study. In Co-Pilot, 12 weeks of combination therapy with the NS3-4A inhibitor ABT-450/r (r = ritonavir-boosted), the NNI ABT-333, and ribavirin resulted in 93 and 47% SVR in treatment-naïve HCV genotype 1 patients and in previous null-responders to pegIFN-α and ribavirin alone, respectively (Poordad *et al*, 2013). Furthermore, in the single arm of the so-called Pilot study evaluating ABT-450/r in combination with the NNI ABT-072 and ribavirin, SVR was achieved in 91% (10/11) of treatment-naïve HCV genotype 1 patients, who, however, all had a good-response *IL28B* CC genotype (Lawitz *et al*, 2012).

Though both NS3-4A inhibitors and NS5A inhibitors are burdened with a low barrier to resistance development, their high antiviral efficacy has encouraged several investigators to combine these agents in IFN-free trials. The first clinical trial which has reported SVR data for an IFN-free regimen investigated the combination of the NS5A inhibitor daclatasvir (BMS-790052) with the NS3-4A protease inhibitor asunaprevir (BMS-60032) for 24 weeks in 11 HCV genotype 1 patients with prior null-response to pegIFN-α and ribavirin (Lok *et al*, 2012). Four of these 11 patients (36%) achieved SVR (2 of 9 genotype 1a, 2 of 2 genotype 1b). Viral breakthrough was observed only in patients infected with HCV genotype 1a, and in all of them resistance mutations against both agents were detected. Nevertheless, this trial constituted a proof-of-principle that SVR can be achieved by all-oral regimens, especially in patients infected with HCV subtype 1b. These principal findings have been confirmed by larger follow-up studies, leading to an ongoing phase 3 trial of daclatasvir and asunaprevir in genotype 1b patients. More promising data for genotype 1a patients have been presented for an all-oral regimen including the NNI BMS-791325 added to daclatasvir and asunaprevir (see below).

#### Combinations of multiple DAAs with or without ribavirin

A multiple DAA regimen containing the NS5A inhibitor ledispavir (GS-5885), the NS3-4A inhibitor GS-9451, the NNI tegobuvir, and ribavirin (QUAD study) yielded SVR rates of 77 and 89% in treatment-naive HCV genotype 1a and 1b patients, respectively (Sulkowski *et al*, 2012b). However, this all-oral quadruple regimen was restricted to approximately 70% of patients in whom HCV RNA felt below the limit of detection until treatment-week 2, whereas the remaining patients were switched to a PEG-IFN-α-based rescue therapy. The AVIATOR study evaluated an all-oral quadruple therapy included the NS3-4A inhibitor ABT-450/r, the NS5A inhibitor ABT-267 and/or the NNI ABT-333, plus ribavirin for 8–24 weeks. In both treatment-naïve patients and prior null-responders with HCV genotype 1 infection, combination of three DAAs plus ribavirin led to comparable SVR rates after 12 or 24 weeks of treatment (higher than 90%), whereas slightly higher relapse rates were noted after 8 weeks of therapy (Kowdley *et al*, 2013b). Overall, 12-week duration of this regimen appeared favourable and was well tolerated. Highly promising results were also obtained for the IFN-free combination of the NS3-4A inhibitor asunaprevir in combination with the NS5A inhibitor daclatasvir and the NNI BMS-791325 for 12–24 weeks. This regimen yielded an overall SVR rate of approximately 90% of treatment-naïve HCV genotype 1 patients, including a significant proportion of HCV subtype 1a patients (Everson *et al*, 2013).

## Interpretation of studies published thus far and perspectives to overcome further challenges

Shortly after the discovery of HCV in 1989, IFN-α monotherapy was approved for the treatment of HCV infection. With the approval of telaprevir- and boceprevir-based triple therapies with PEG-IFN-α and ribavirin in 2011, chances of cure for patients infected with HCV genotype 1 have improved from <10% to about 70%. Limitations of telaprevir- and boceprevir-based triple therapy are limited efficacy against HCV genotypes 2–6, significant side effects and drug-drug interactions, and low success in patients with advanced liver disease and previous null-response to PEG-IFN-α/ribavirin therapy. Triple therapies based on some next-generation NS3-4A protease inhibitors, selected NS5B inhibitors, or NS5A inhibitors were shown to (partially) overcome these limitations. Yet, the triple therapy approach has important intrinsic shortcomings. Most importantly, the IFN-α/ribavirin backbone, which is necessary to avoid resistance development during treatment with these regimens, is responsible for a low efficacy of triple therapy in prior null responders to PEG-IFN-α and ribavirin. Furthermore, patients with contraindications to PEG-IFN-α or ribavirin cannot be treated with triple therapy. The latter applies also to DAA combinations in PEG-IFN α/ribavirin-based quadruple treatment regimens, which, however, appear to be very potent even for difficult-to-cure patient populations such as HCV genotype 1a patients with prior-null response. With respect to the changing epidemiology of HCV infected populations, numerous HCV infected patients who require antiviral therapy will present with advanced liver disease and higher age in the very near future. Therefore, tolerability will represent an important feature of HCV therapy and—though successful in most patients who tolerate quadruple therapy—the quadruple approach will probably become a first-choice therapy for only a minority of patients, if at all. Hence, IFN-free all-oral regimens will certainly become a first choice for a significant number of patients in the very near future, a scenario, which had appeared speculative until very recently. Due to the inconvenience of PEG-IFN-α-based therapies, IFN-free treatment regimens would certainly be preferable also for patients who could be treated with triple or quadruple therapy as well.

Early in the evolution of IFN-free therapies, it appeared that response in patients treated with such regimens was adversely impacted by prior nonresponse to IFN (Poordad *et al*, 2013) or host factors such as “unfavourable” *IL28B* genotypes (Chu *et al*, 2012; Poordad *et al*, 2013). Hence, there arose the concept that an appropriate endogenous immune response against HCV may still be of importance for the final clearance of residual virus In addition to these host-associated determinants of treatment outcome, HCV genotype 1a appeared to be associated with lower rates of SVR to both triple therapy and all-oral therapy based on either NS3-4A inhibitors or NS5A inhibitors. Nevertheless, recent studies have clearly shown that the combination of different potent DAAs (*e.g*. the NI sofosbuvir plus the NS5A inhibitor daclatasvir or multiple DAA combinations) can sufficiently overcome these limitations and have the capacity to cure the vast majority even of patients with such unfavourable baseline characteristics. Thus far, it appears that regimens without a component conferring a high barrier to resistance (such as an NI) may require 1–2 additional drugs to optimize response across patient populations and HCV genotype 1 subtypes. Optimization of IFN-free therapy in genotype 3 may ultimately require “second generation” NS5A or NS3/4A inhibitors that have pangenotypic activity, and other genotypes, including genotype 4, require further study. The ultimate goal in the development of hepatitis C therapies, a single-(or minimal) pill regimen with few side-effects, limited drug-drug interactions, and the capacity to cure almost all HCV-infected individuals (including liver transplanted patients), independently from previous treatment outcome, infection with distinct HCV genotypes/subtypes, coinfections (HBV, HIV), or the stage of liver disease, could be achieved in the very near future.

Based upon the findings described in this review, pivotal studies of all-oral combinations, including patients with cirrhosis, have been initiated. The high costs of some novel treatment regimens and consequences for countries with limited resources may require attention. Furthermore, progress in the treatment of chronic hepatitis C should not distract researches from other urgent needs, *e.g*. in order to minimize the number of individuals with undiagnosed hepatitis C. Finally, treatment-induced eradication from HCV infection results in significant reduction of HCV-related morbidity (van der Meer *et al*, 2012), but even cured patients require attention by physicians as they may remain burdened with higher mortality than individuals who were never infected with HCV, especially those with advanced fibrosis (Innes *et al*, 2011).

## For more information

http://www.aasld.org; http://www.easl.eu

(these associations provide current American and European clinical practice

guidelines for the treatment of HCV infection).

http://www.hepatologytextbook.com

(a free medical textbook which provides a comprehensive and up-to-date

overview of all aspects of liver diseases including hepatitis C).

## Abbreviations

Cyclophilin A: is a peptidyl-prolyl-isomerase, which is mandatory for proper folding of several proteins. Inhibition of cyclophilin A by calcineurin inhibitors inhibits T cell activation and identifies cyclophilin A as an important target to suppress auto- and alloimmunity.
Decompensation of liver cirrhosis: is a general term indicating the presence of certain acute or chronic complications of liver cirrhosis. Such complications include for example the development of ascites, hepatic encephalopathy, spontaneous bacterial peritonitis or bleeding due to liver-related coagulopathy/portal hypertension.
In GWAS: (genome-wide association studies) genotypes of up to millions of single nucleotide polymorphisms (SNPs) throughout the entire genome are determined and compared between groups of individuals, which differ with respect to a defined phenotype. In contrast to candidate gene studies, the random approach of GWAS allows the identification of unknown and unexpected genetic variants associated with the respective phenotype.
MAVS: (mitochondrial antiviral-signaling protein) is an adaptor protein in the RIG-I (retinoic acid inducible gene I) signaling pathway. RIG I senses double stranded viral RNA, which leads to binding of MAVS and subsequent activation of the transcription factors interferon regulatory factor 3 (IRF3) and NF-κB, which are key players in innate antiviral immunity.
Ritonavir: is an HIV protease inhibitor, which inhibits cytochrome P450 and thereby increases serum concentrations of drugs, which are metabolized by cytochrome P450. Therefore, co-administration of ritonavir (ritonavir-boosting) is applied to increase bioavalability of some drugs including several HCV protease inhibitors.
Sustained virologic response (SVR): is defined as undetectable HCV RNA 24 weeks after antiviral therapy. SVR is generally considered as cure from HCV infection, which reduces but not completely eliminates liver-related morbidity in HCV patients.
Subgenomic HCV replicons: are RNAs in which parts of the genome encoding the structural (and in some cases parts of the non-structural) HCV proteins are replaced by genes allowing the selection of replicons for example during exposure to antibiotics. In human hepatoma cell lines, subgenomic replicons replicate and HCV polyprotein translation occurs, but no viral particle production is possible due to the lack of structural proteins
